# Melting curve of SiO_2_ at multimegabar pressures: implications for gas giants and super-Earths

**DOI:** 10.1038/srep26537

**Published:** 2016-05-23

**Authors:** Felipe González-Cataldo, Sergio Davis, Gonzalo Gutiérrez

**Affiliations:** 1 Departamento de Física, Facultad de Ciencias, Universidad de Chile, Casilla 653, Santiago, Chile.; 2 Comisión Chilena de Energía Nuclear, Casilla 188-D, Santiago, Chile.

## Abstract

Ultrahigh-pressure phase boundary between solid and liquid SiO_2_ is still quite unclear. Here we present predictions of silica melting curve for the multimegabar pressure regime, as obtained from first principles molecular dynamics simulations. We calculate the melting temperatures from three high pressure phases of silica (pyrite-, cotunnite-, and Fe_2_P-type SiO_2_) at different pressures using the Z method. The computed melting curve is found to rise abruptly around 330 GPa, an increase not previously reported by any melting simulations. This is in close agreement with recent experiments reporting the *α*-PbO_2_–pyrite transition around this pressure. The predicted phase diagram indicates that silica could be one of the dominant components of the rocky cores of gas giants, as it remains solid at the core of our Solar System’s gas giants. These results are also relevant to model the interior structure and evolution of massive super-Earths.

Melting is the major force in chemical differentiation of the Earth, and plays a fundamental role in the physical and chemical evolution of planetary interiors. Knowing the melting temperature at high pressures of end-members—silicates, iron, iron alloys, and H_2_O—is needed to better constrain the internal structure and physical properties of super-Earths, defined as those planets with masses between 1 and 10 Earth masses. These extrasolar planets achieve large pressures, up to ~60 Mbars where current equations of state are being extrapolated without any experimental data to constrain them.

The quest for extrasolar planets has also revealed hundreds of worlds of different classes, inspiring novel concepts about planetary formation and composition. The discovery of super-Earths and extrasolar giant planets has provided enormous datasets of their atmospheric composition, size and mass, and has opened new possibilities for understanding planetary formation and composition^1^,^2^,^3^. As the occurrence of discovered super-Earths increases, and precision improves, high-pressure physics becomes more relevant for determining the properties of these planets. The large-scale interior processes of planets are controlled by the physical properties of the mineral constituents, which in turn are controlled by their underlying chemistry and crystal structure. Thus, the study of thermodynamic properties of materials at high pressure, such as melting, provides a sympathetic insight of the interior of such planets^4^.

Planets appear to be far more diverse than previously thought, and knowledge of the mineralogy of super-Earth planets is essential for understanding their interior structure, thermal behavior, and long-term evolution. The mineralogy will depend on bulk composition and the pressures and temperatures of the interior. The large sizes of super-Earth planets mean that interior pressures are very high, posing a severe challenge for laboratory experiments attempting to reproduce super-Earth conditions^5^. Models for the interior of planets have been developed through the years^6^,^7^,^8^,^9^ based on astronomical and condensed matter knowledge, and are essential for interpreting observations of their masses, radii, and atmospheres, and for understanding the physicochemical diversity of planetary systems. These models depend deeply on our knowledge of the properties of rock-forming elements^10^,^11^,^12^,^13^, such as perovskites and silicates. The estimation of the planetary internal composition is based on a mass to radius relation that relies heavily on equations of state (EOS), a relationship between the pressure, temperature and density of a material in thermal equilibrium. The EOS is one of the most important high-pressure material properties, which can be calculated from electronic structure theory or measured experimentally on Earth, and it is an essential component of interior structure models for exoplanets. Thus, studying the properties of the end-member oxides is an important starting point to understand solid solution, solubility, and dissociation reactions in plausible mineral assemblages. Giant planets resemble natural laboratories for studying the behavior of materials at high pressure and temperature, which typically reach values outside the realm of experiment. Therefore, theoretical approaches, such as ab initio simulations, provide the best available guide to study the properties of matter at such extreme conditions^1^,^14^,^15^. The role that they play, together with high-pressure experiments, is crucial in determining super-Earth properties^4^.

While some constraints on melting curves and liquid properties from theory and experiment are available for some of the materials discussed earlier, there is still considerable uncertainty in many fundamental properties, including solidus temperature, melt-solid density contrast, and partitioning behavior, at pressures of 100–1000 GPa and beyond. Calculating the melting curves of rock-forming materials is of particular importance to predict whether the lowermost mantles of super-Earths can easily melt, or not^9^, and to determine if there are magma reservoirs in the mantle^8^. The melting temperature of perovskite, for instance, is probably always more than 1000 K above the Earth’s geotherm, and this difference increases with pressure^16^. This feature is more pronounced in super-Earths, such as CoRoT-7b, since the pressure here increases more rapidly with depth than on Earth, which has been important in determining if temperature is high enough to allow stability of silicates in solid state inside this planet^8^. However, melting curves on other end-members are also necessary to have a broader picture.

The most abundant oxide component of terrestrial mantles is silica, a prototype for the dense, highly coordinated silicates of planetary interiors. Commonly found in meteorites^17^, silica, together with other silicates such as MgSiO_3_ and CaSiO_3_, are the major components of the Earth’s lower mantle. While free SiO_2_ is only housed in localized regions of the Earth’s mantle, such as subducted oceanic crust, the higher *P*-*T* conditions and expanded range of plausible compositions in super-Earth exoplanets allow for greater possible presence of silica phases in terrestrial exoplanets, thus forming the bulk composition of super-Earths^5^,^9^,^18^,^19^ as well as the rock-ice protocores that result in gas giants through core accretion^20^. It has been determined that MgSiO_3_ dissociates in the cores of gas giants and terrestrial exoplanets^21^,^22^, leaving SiO_2_ and MgO as important separate compounds that form the rocky interior of these planets. In this context, the phase diagram of silica becomes fundamental. Currently, the melting curve for silica is known only up to 500 GPa (5 Mbar)^23^ by means of shock experiments, but no static pressure experiments have been able to reach these conditions. Simulations have reported the melting curve up to 200 GPa^24^, and recently up to 3400 GPa^25^, both using the two phase method. Since the multimegabar regime corresponds to the conditions found in the core of giant planets and super-Earths^21^, it is desirable to explore it for even higher pressures, and from different approaches.

Changes in crystal structure can affect the melting temperature, therefore melting from different structures must be considered. SiO_2_ goes through a series of phase transitions as the pressure increases: quartz → coesite → stishovite → CaCl_2_-type → *α*-PbO_2_-type → pyrite-type. Post-pyrite structures depend on temperature and have been identified using ab initio calculations^21^,^26^,^27^,^28^. According to these calculations, at low temperature the transition from pyrite to Fe_2_P would occur near 700 GPa. Above 1000 K, silica first transforms from the pyrite-type structure into cotunnite and then into Fe_2_P. Recent ab initio calculations^24^ of the melting curve of SiO_2_ up to 160 GPa (using the two-phase molecular dynamics method) show good agreement with previous calculations, which were done with the same method but using classical potentials^29^,^30^. However, the solid-liquid boundary for silica at higher pressures remains unknown.

In this work we extend the melting curve of SiO_2_ up to 6000 GPa (60 Mbar), covering the range of pressures and temperatures that exist at the interiors of gas giants and massive super-Earths. The melting curve of SiO_2_ at these ultra-high pressures was obtained by using first principles molecular dynamics (FPMD) simulation, together with the Z method and Bayesian statistics. Three phases of silica are considered: pyrite, cotunnite and Fe_2_P.

## Results

The melting curve was obtained in the following way. Using the Z method, we obtained several (*P*_*m*_, *T*_*m*_) melting points (where *T*_*m*_ is the melting temperature at the pressure *P*_*m*_) from the three structures considered: 7 from the pyrite structure, 8 from the cotunnite structure, and 7 from the Fe_2_P structure. It is known that the structure with the highest melting temperature is the more stable phase^31^,^32^; then, the melting curve corresponds to the collection of the melting points that have the highest melting temperature for a given pressure. This curve is also known as the *solidus* of the material, which separates the regions of pressures and temperatures at which silica is completely solid from those in which partial or complete melting takes place.

In order to obtain a continuous melting curve, the melting points obtained were fitted to a Simon melt equation as follows:


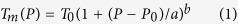


where *P* is the applied pressure, *T*_*m*_(*P*) the melting temperature corresponding to that pressure, and the values of the adjustable parameters are *T*_0_ = 6551.246 K, *P*_0_ = 317.068 GPa, *a* = 116.7226 GPa and *b* = 0.2966. This fit describes the portion of the curve greater than *P*_0_, since this expression cannot describe a discontinuity between phases that we will refer to in the next paragraphs. Millot *et al.*^23^ proposed a fit to their experimental data of the form *T*_*m*_(*P*) = 1968.5 + 307.8*P*^0.485^. From our data, we obtain *T*_*m*_(*P*) = 1968.5 + 478.7*P*^0.426^, which shows very good agreement with the experiments.

**Figure 1:**
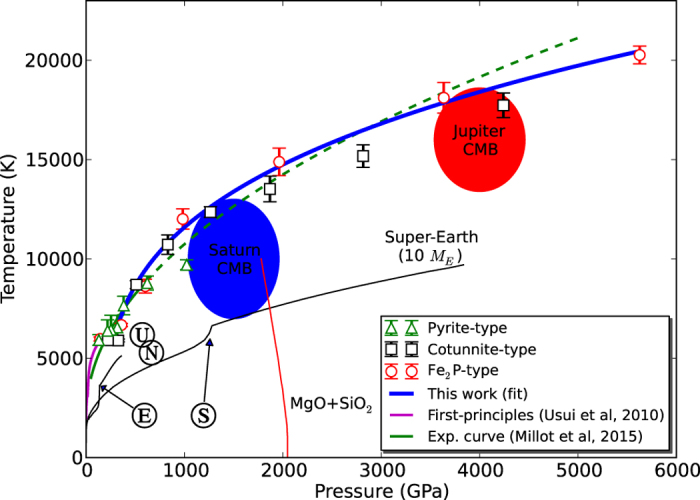
Melting data of SiO_2_, along with *P*-*T* conditions predicted for the interior of rocky planets of 1 and 10 Earth masses. Green triangles, black squares and red circles represent the melting conditions for pyrite, cotunnite and Fe_2_P phases, respectively. Error bars indicate the uncertainty in the melting temperature computed by the Bayesian procedure^44^. The melting curve of silica is obtained by fitting our data (thick, blue line, Equation (1)) using the Simon fit equation up to 6 TPa. The blue and red shaded areas correspond to the core-mantle boundary conditions (CMB) Saturn and Jupiter, respectively^1^,^33^. CMB for Earth (E), a super-Earth (S), Uranus (U)^61^ and Neptune (N)^61^ are shown for comparison. The predicted dissociation of MgSiO_3_ post-perovskite into MgO and SiO_2_ is shown in red^21^,^22^. The melting curve of SiO_2_ at the highest pressures available to date, obtained from two-phase MD simulations^24^ (magenta) and from experiments up to 500 GPa^23^ (in green, and dashed when extrapolated), are also shown. The black lines stand for Super-Earth (10 *M*_*E*_) and Earth (1 *M*_*E*_) internal pressure-temperature ranges obtained from planetary models^5^,^6^.

**Figure 2:**
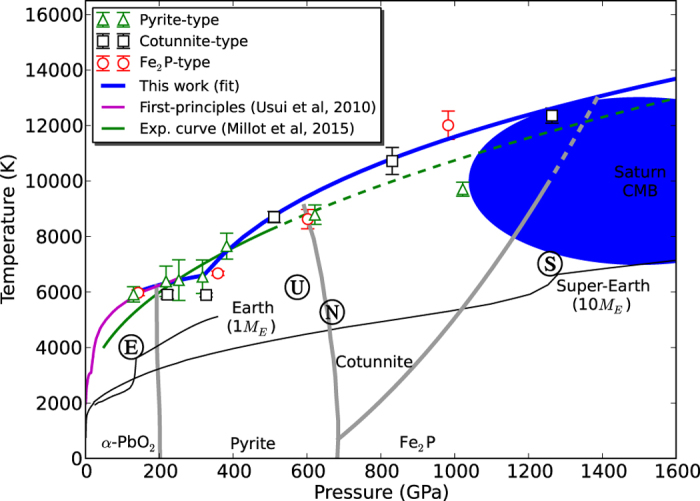
Details of SiO_2_ melting curve, along with solid-phase boundaries. Symbols are the same as in Fig. 1. Gray lines are predicted solid-phase boundaries from ref. 26 and 27 (dashed when extrapolated). The melting curve of SiO_2_ obtained from shock experiments^23^ and from two-phase simulations^24^ are shown in green and magenta, respectively (dashed when extrapolated).

In Fig. 1 we show all the obtained melting points along with the melting curve of SiO_2_ up to 6000 GPa. The symbols represent the calculated melting point for pyrite (triangles), cotunnite (squares) and Fe_2_P (circles). Error bars indicate the uncertainty in the melting temperature. At low pressure (~300 GPa, see Fig. 2), pyrite points are above the other two phases. Then, at slightly higher pressure (~500 GPa), cotunnite melting temperatures overcomes pyrite temperatures. Finally, in the high end pressure range, Fe_2_P points are above the others. In order to draw the solid–liquid boundary (blue line in Fig. 1), we chose the more stable phase, that is, the structure corresponding to the higher melting temperature at a given pressure.

The calculated melting curve covers the range of pressures and temperatures relevant to the interior of super-Earths and giant planets, and provides constraints for the overall thermal state of planetary mantles. Although the pressures present at the Earth’s deep interior are about a few megabar (~300 GPa), super-Earths can overcome this limit and have a core-mantle boundary pressure higher than 1000 GPa^6^,^7^ (see Fig. 1). In the case of giant planets, the pressure reached at the core-mantle boundary can exceed 4000 GPa^1^, and knowing the melting temperature of silica at these pressures provides guidance for further studies, such as the calculation of the solubility of silica in metallic hydrogen^33^. Our results show that the silica melting curve lies above the core-mantle boundary of the giant planets of our solar system and above the core-mantle boundary of Earth, Uranus, Neptune, and super-Earths. Thus, silica is expected to be in solid state at the very interior of these planets.

Figure 2 shows in detail the 0–1600 GPa pressure region of Fig. 1. We observe a very good agreement with the calculations performed by Usui and Tsuchiya^24^, who used two-phase simulations to obtain the melting curve up to 160 GPa. We also observe that a discontinuity in the slope of the curve occurs at 330 GPa, regardless of the initial structure from which silica is molten (pyrite, cotunnite and Fe_2_P melting points rapidly rise from the 200–300 GPa plateau observed in the melting curve), suggesting that the *α*-PbO_2_–pyrite transition would take place at 330 GPa, and not around 200 GPa as suggested by previous simulations^26^, which is the phase boundary shown in Fig. 2. Our result is much closer to the experiments, which suggests a transition around 290 GPa^34^.

Fitting the pyrite melting points and the cotunnite melting points, we can build the pyrite and cotunnite solidus, and these curves intersect at 480 GPa. Therefore, the picture that emerges from our results is that the stability region of pyrite extends up to 480 GPa, not far from the predicted 550 GPa obtained by recent simulations^27^,^28^. We believe that the difference is due to the validity of the quasi-harmonic approximation used to determine these boundaries. Considering the Clausius–Clapeyron equation *dP*/*dT* = Δ*S*/Δ*V*, and taking into account that at high pressure Δ*S* ≈ *k*_*B*_ ln 2^35^, the change in the slope of the melting curve at around 480 GPa is indicative of a volume reduction of Δ*V* ≈ 0.114 Å^3^ per SiO_2_ unit. The stability of the cotunnite phase ends at 900 GPa, where the Fe_2_P type phase begins. Thus, our simulations suggest that the boundaries of the cotunnite phase occurs between 480 and 900 GPa and that at higher pressures, the stable phase is Fe_2_P. A previous study by Tsuchiya *et al.*^27^ used MD and metadynamics simulations to estimate the pyrite-cotunnite boundary at low temperature around 650 GPa. The extension of this boundary to high temperature intersects with our melting curve at 600 GPa (Fig. 2), generating a pyrite–cotunnite–liquid triple point. An extrapolation of their cotunnite-Fe_2_P boundary hints at another triple point around 1400 GPa. However, according to our results, both triple points occur at lower pressures than predicted by Tsuchiya *et al.* This suggests a displacement of both solid-solid boundaries, but as in the case of Tsuchiya *et al.*, it also confirms that the stability range of the cotunnite phase increases with increasing temperatures.

## Discussion

Although we have only considered homogeneous melting, our reported melting curve places a lower limit of stability for solid silica structures. Above this limit, the crystalline structure becomes unstable and nucleation can take place to initiate melting. Inclusion of impurities is not considered in this work, but their effect in the melting temperature of silica definitely deserves a separate study. A comparison of the melting curves of other end-members such as MgO, perovskites and iron alloys would be very useful to predict whether the interiors of gas giants consist of stable rocky cores. Although the MgO melting curve remains unknown for pressures higher than 600 GPa, its melting temperature is always higher than that of silica up to this pressure^36^, and it has been reported that no further solid-solid transitions take place up to 4000 GPa^37^. Therefore, it is quite possible that the MgO melting curve may continue to increase monotonically, staying always above the SiO_2_ melting temperature.

Our results have important implications for the interior of super-Earths. The interior of supermassive rocky planets, such as CoRoT-7b, CoRoT-3b, Kepler-10b and GJ 1214b, exhibit pressures that can easily exceed 1000 GPa^15^,^38^, where magnesium perovskite dissociation is promoted and the properties of MgO and SiO_2_ become relevant. We predict that the SiO_2_ component will remain as a stable solid component in the deep interior of super-Earths -deep enough to reach very high pressures, but not reaching the CMB- as a temperature greater than 10000 K is needed to melt it beyond 700 GPa. This temperature is too high for the interior of a rocky planet^5^,^6^. The greater possible presence of silica phases in terrestrial exoplanets augments the importance of its phase diagram. Along the possible *P*-*T* path for a super-Earth, the pyrite-cotunnite transformation is expected to occur for planets of approximately 5 *M*_*E*_ or greater. The cotunnite-Fe_2_P transformation would be expected in planets of about 7.5 *M*_*E*_^5^. These phase transitions will affect the density profile of the planet, and the melting of silicates will regulate planetary evolution through heat flow. Since planets with mantles have hotter interiors due to their insulating character, the knowledge of the melting point of rocks becomes crucial. Studies about the melting behavior of post-perovskite and MgO at these pressures must be performed since, coupled with our results, this could finally clarify if the lower-most mantle of super-Earths can easily melt, or not^9^,^18^,^39^. In addition, this could yield valuable information for the study of plate tectonics, where the inclusion of melts significantly impacts cooling and modulates the thermal evolution^40^,^41^.

Formation of magma oceans is also dependent on the melting properties of components, like silica. The difference between the melting temperature of silicates and the geotherm of a planet becomes more significant in planets with high surface gravity, since there is a more rapid increase in pressure with depth than on Earth, as in CoRoT-7b, where the surface gravity is twice as much as it is on Earth^8^. The abrupt increase in the slope of the SiO_2_ melting curve obtained in this study confirms the assumption that the temperature through the mantle is not high enough to allow the silicates to be stable in a liquid state at depth if they are not already molten at the surface. As a consequence, the existence of underground oceans, or even large magma reservoirs, is unlikely on planets like CoRoT-7b^8^. However, larger rocky exoplanets, like Kepler 20-b or 55 Cnc e, would likely have an extended basal magma ocean in the lower mantle that could affect their magnetic field and reinforce tidal response^23^.

On the other hand, and according to the most recent models, the conditions at the interior of giant planets lie in the megabar to gigabar pressure range, at temperatures on the order of 10^4^ K^14^,^21^,^33^,^39^,^42^ which, according to our study, is just the region where silica is expected to melt. This is an interesting scenario, since the rocky core of gas giants with interiors hotter than Jupiter, but with similar pressures at the core–mantle boundary, may partially melt their core, with substantial implications in the evolution of the planet; while larger gas giants, with higher inner pressures, should at least have a solid silica component. In the case of the gas giants of our Solar System, and contrary to what other works have concluded^25^, based on a melting line that goes well below the extrapolated experimental curve^23^ and different core–mantle boundary conditions, we conclude that silica is expected to be in solid state in their core, as shown in Fig. 1, although this does not mean that the core remains stable, since it can be affected by the solvation effect of metallic hydrogen as demonstrated in previous studies^33^. These claims must be put before better constrained models for the interior of these planets in order to solve the controversy, since the pressure and temperature inside Saturn and Jupiter, although constrained to a range, are still quite uncertain. Studies relating the melting temperature with the viscocity^43^, may find the results presented here valuable, since at low pressures the viscosity is thought to be proportional to the difference between the geotherm and the solidus (so called homologous viscosity model) so that a super-Earth mantle with temperatures significantly sub-solidus may have high viscosity, and may not be fully convective. However this is purely speculation with only theoretical predictions of super-Earth viscosities and no experimental constraints. This should be pursued in the future.

## Conclusions

In summary, we have presented the SiO_2_ melting curve by using ab initio calculations and the Z method, up to 6000 GPa and 20000 K. The results allow us to determine the solid-solid boundary between high pressure silica phases at the melting point. Previous calculations of the melting curve of silica at high pressures, performed with the two-phase method, have only reached 160 GPa, and our results show very good agreement with them. We estimated the pyrite-cotunnite phase transition at 480 GPa, and the cotunnite-Fe_2_P at 900 GPa along the melting curve. According to our results, silica, if present, is solid within the cores of all Solar System gas giants and, together with MgO, is a key component of the stable rocky cores of extrasolar gas giants. Therefore, we expect the calculated SiO_2_ melting curve to be useful in the study of planetary interior models.

## Method

We obtain the melting curve of SiO_2_ using the Z method, which is a procedure that has been extensively used in multiple melting studies^31^,^32^,^44^,^45^,^46^,^47^,^48^,^49^,^50^,^51^,^52^,^53^,^54^,^55^,^56^,^57^,^58^,^59^,^60^, even in materials with anomalous melting curves, such as Li and H. The idea is to perform FPMD in the microcanonical ensemble (NVE) on a single solid system at different initial total energies. The total energy is controlled by setting a different initial temperature in each simulation. When the crystal is heated beyond its overheating limit, the temperature naturally drops to the melting temperature as the latent heat is removed from the kinetic energy. The connected *P*-*T* points on the isochore form a Z-shaped curve. Several simulations for each isochore are needed in order to yield an accurate melting temperature.

We perform Z method simulations of SiO_2_ melting with the Vienna ab initio simulation package (VASP) for pyrite (for seven volumes, 11.06 Å^3^/f.u., 13.01 Å^3^/f.u., 15.17 Å^3^/f.u., 16.10 Å^3^/f.u., 17.07 Å^3^/f.u., 17.57 Å^3^/f.u. and 20.20 Å^3^/f.u., where f.u. denotes a formula unit of SiO_2_), cotunnite (for eight volumes, 6.49 Å^3^/f.u., 7.63 Å^3^/f.u., 8.90 Å^3^/f.u., 10.31 Å^3^/f.u., 11.85 Å^3^/f.u., 13.54 Å^3^/f.u., 15.38 Å^3^/f.u. and 17.39 Å^3^/f.u.), and Fe_2_P (for seven volumes, 5.79 Å^3^/f.u., 6.95 Å^3^/f.u., 8.81 Å^3^/f.u., 11.31 Å^3^/f.u., 13.10 Å^3^/f.u., 15.06 Å^3^/f.u. and 19.55 Å^3^/f.u.). For each volume, a number of initial temperatures are adopted, ranging from 5000 to 50000 K with the interval of 1000~5000 K. To obtain more accurate melting temperatures, we have coupled the Z method with a Bayesian statistical analysis following the work of Davis *et al.*^44^. The details of this analysis are explained in the supplementary material.

The FPMD is based on the density functional theory (DFT) in the Born–Oppenheimer approximation. We used PAW pseudopotential, and the Perdew-Burke-Ernzerhof (PBE) exchange-correlation functional. For the plane wave expansion of the wavefunctions, we used a cutoff energy of 900 eV, and a 1 × 1 × 2 k-point grid to sample the Brillouin zone in the case of cotunnite-type SiO_2_, and Γ–point only for the pyrite and Fe_2_P structures. The simulation cells are constructed with 96 atoms for cotunnite and pyrite structures, and with 72 atoms for Fe_2_P. For all simulations, the time step chosen was 0.2 fs, such that the energy drift is negligible. The simulation time was chosen to be 5000 steps, which gives enough time for the pressure and temperature to converge, and gives a melting temperature that is corrected by the Bayesian analysis mentioned above. Additional convergence tests for the particle number are performed by repeating an isochore of 8.81 Å^3^ on a 243-atoms cell containing the Fe_2_P structure. Simulation time convergence was also tested by performing 30000 time steps (6 ps) simulations close to the melting temperature on the pyrite structure. No changes in the average properties were observed after performing these tests.

## Additional Information

**How to cite this article**: González-Cataldo, F. *et al.* Melting curve of SiO_2_ at multimegabar pressures: implications for gas giants and super-Earths. *Sci. Rep.* **6**, 26537; doi: 10.1038/srep26537 (2016).

## Supplementary Material

Supplementary Information
