# Supplementary material for: Melting curve of SiO2 at multimegabar pressures: implications for gas giants and super-Earths
Source: Sci Rep. 2016 May 23;6:26537. doi: 10.1038/srep26537 (PMC4876395; doi:10.1038/srep26537)
Supplement: Supplementary Information [file srep26537-s1.pdf]

Melting curve of SiO_2 at multimegabar pressures: implications for gas giants and super-Earths: Supplementary material

F. González-Cataldo,* S. Davis, and G. Gutiérrez
Departamento de Física, Facultad de Ciencias, Universidad de Chile
(Dated: March 31, 2016)

PACS numbers:

Methodology

As stated in the main part of this report, the melting curve of SiO_2 at ultra-high pressure was calculated using the Z method, an approach that has recently received a wide application in melting studies[1–19]. We apply the method by means of first-principles molecular dynamics (FPMD) simulations based on Density Functional Theory (DFT). Then, a Bayesian statistical analysis, as performed by Davis and Gutiérrez[14], is coupled to the Z method in order to constrain the melting point and its uncertainty from the simulation data. Three phases of pyrite, cotunnite and Fe_2P are considered[20–22], and around eight isochores are calculated for each structure: for pyrite, seven volumes: 11.06 $Å^3$/f.u., 13.01 $Å^3$/f.u., 15.17 $Å^3$/f.u., 16.10 $Å^3$/f.u., 17.07 $Å^3$/f.u., 17.57 $Å^3$/f.u. and 20.20 $Å^3$/f.u., where f.u. denotes a formula unit of SiO_2); for cotunnite, eight volumes: 6.49 $Å^3$/f.u., 7.63 $Å^3$/f.u., 8.90 $Å^3$/f.u., 10.31 $Å^3$/f.u., 11.85 $Å^3$/f.u., 13.54 $Å^3$/f.u., 15.38 $Å^3$/f.u. and 17.39 $Å^3$/f.u.); and for Fe_2P, seven volumes: 5.79 $Å^3$/f.u., 6.95 $Å^3$/f.u., 8.81 $Å^3$/f.u., 11.31 $Å^3$/f.u., 13.10 $Å^3$/f.u., 15.06 $Å^3$/f.u. and 19.55 $Å^3$/f.u.). For every volume, a number of temperatures are adopted, ranging from 5000 to 50000 K with the interval of 1000 $\sim$ 5000 K. We used supercells containing 72 atoms for the Fe_2P structure, and 96 atoms for the pyrite and cotunnite structures. Due to the high temperatures and pressures used, the atoms move faster than in room conditions and the time step must be reduced accordingly, in order to minimize numerical errors and ensure the conservation of total energy. The period of oscillation of individual atoms can be estimated from the first maximum of the velocity autocorrelation function (VACF), corresponding to approximately 30 fs for both Si and O (see Fig. S1). Thus, a time step of 0.2 fs, as also used in previous studies at similar conditions[5,23–27], was small enough to resolve the fine details of atomic movement and to make any energy drift in the NVE simulations negligible. This time step was used in all simulations. We equilibrated the structures for 5000 steps (1 ps), which we found was enough to get a defined slope in the liquid and solid regions.

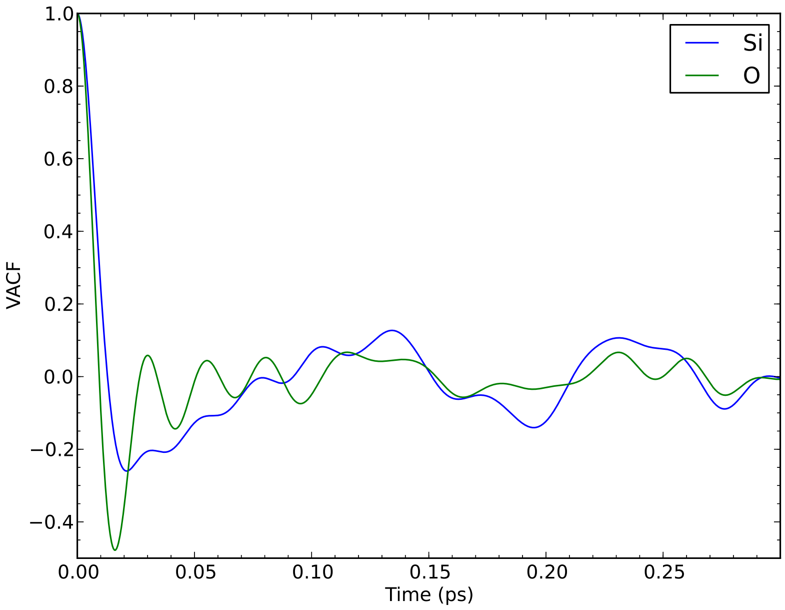

FIG. S1: Velocity autocorrelation function (VACF) of SiO_2 in the pyrite structure at $\sim$ 7000 K and density $\rho = 6.58$ g/cc ($\sim$ 340 GPa). The first maximum corresponds to the time scale of the period of oscillation of individual atoms.

S1. Z-METHOD

In atomistic computer simulation in the microcanonical ensemble it is relatively easy to overheat a crystal beyond its melting temperature T_m. This puts the system in a metastable state (the superheated solid state) which survives until the final equilibrium temperature reaches a limit T_{LS} known as the critical superheating limit[2]. Once beyond this limit, the superheated state is unstable and melting will spontaneously ocurr during the simulation (if long enough times are used).

For a temperature of the superheated phase T_{LS}^{+} (slightly above T_{LS}) the equilibrium temperature after melting will drop to T_m^{+} (slightly above T_m). A good estimation of T_m can thus be obtained if one searches for the lowest T that triggers melting during the simulation and computes the final equilibrium temperature. As it has been observed[1–19], the system can transit from the superheated state at T_{LS}^{+} to the liquid state at T_m^{+} at constant energy and volume; thus it is verified

$$E_S(T_{LS}; V) = E_L(T_m; V) \tag{S1}$$

where E_S and E_L are the energies of the solid and liquid branches, respectively, as a function of temperature and volume. We denote the common energy in Eq. S1 as the energy of melting $E_S(V)$ for a given volume V.

The procedure for the Z method computation of the melting point is then as follows: at a fixed volume V, the $(E, T(E))$ points from different simulations draw a "Z" shape, as shown in figure S2, hence the name of the method. Here the states at T_{LS} and T_m have the same energy $E_{LS} \equiv E_S(T_{LS}, V) = E_L(T_m, V)$.

Note that one also can draw the isochore in the pressure–temperature plane $(P(E), T(E))$, as is shown in figure S3. In these Z-shaped curves the sharp inflection at the higher temperature corresponds to $T_{\rm LS}(V)$ and the one at the lower temperature to $T_{\rm m}(V)$. Thus, by computing the temperature T_m and pressure P_m of the lower inflection point for different volumes one obtains an estimate of the melting curve $T_m(P_m)$ for a particular range of pressures.

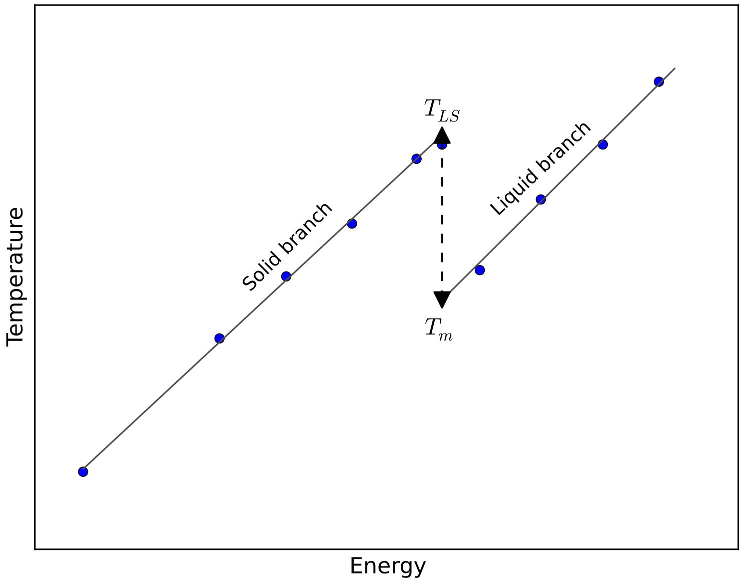

FIG. S2: Schematic representation of the isochoric lines in Z-method simulations in the energy–temperature plane.

In practice, several microcanonical simulations are needed, each with a different initial kinetic energy applied to the ideal crystalline structure with a fixed volume. Each simulation at energy E_i will add a new (E_i, T_i) point to the energy–temperature isochore at volume V (equivalently to the pressure–temperature isochore). With enough points, we obtain isochores in the T-P and T-E diagram which are Z-shaped, as shown in figure S3. However, we need to make sure that the point (T_m, P_m) is in fact the lowest in temperature for the liquid branch, and this can take a considerable number of runs. One possible test is to verify that our estimate of T_m is aligned in energy with our highest estimate of T_{LS}.

It is possible to take advantage of advanced statistical methods to improve the estimations of T_{LS}, T_m and E_{LS} from a number of microcanonical simulations, i.e., statistically reconstruct the most probable isochore given a set

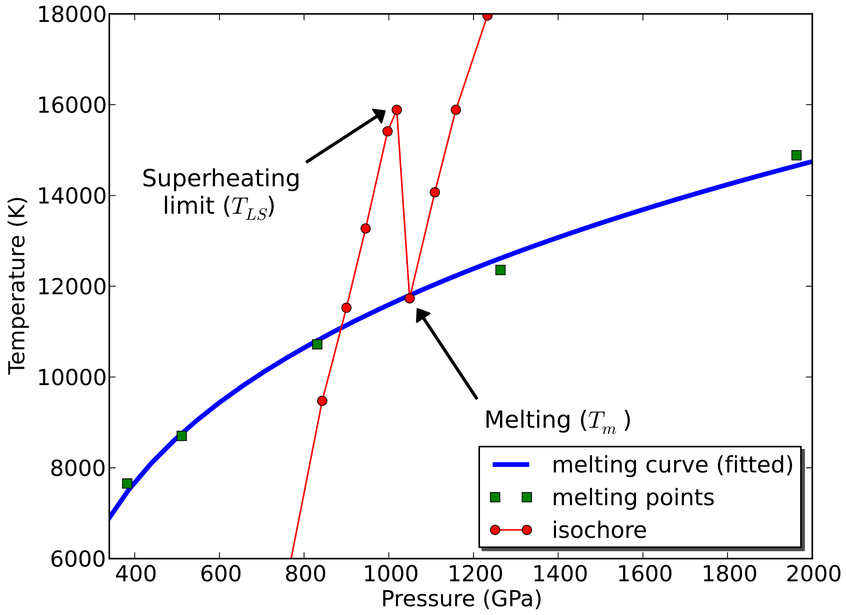

FIG. S3: Example of an isochore in Z method, used to determine the melting temperature in the microcanonical ensemble. The left branch correspond to simulations of the system in solid state, and extends up to the superheating temperature T_{LS}, where the system has an energy E_{LS}. Then, with an energy slightly higher, the equilibrium temperature drops to the melting temperature T_m. The thick blue line represents the actual SiO_2 melting curve calculated in this work.

of points. For a small number of points, simple least squares fitting discards considerable information which can be included by instead implementing a Bayesian estimation procedure, described below.

S2. AB INITIO MD SIMULATION

The FPMD simulations are in the framework of Kohn-Sham density functional theory. We worked under the NVE-ensemble, using the Born-Oppenheimer molecular dynamics (BOMD) method, as implemented in the VASP code[28], employing PAW pseudopotential[29], and the Perdew-Burke-Ernzerhof (PBE) exchange-correlation functional[30]. For the plane wave expasion of the wavefunctions, we used a cutoff energy of 900 eV, and a $1 \times 1 \times 2$ k-point grid to sample the Brillouin zone in the case of cotunnite-type SiO_2, and Γ–point only for the pyrite and Fe_2P structures, as used in previous studies[5].

We checked sistem size dependence, repeating calculations for the Fe_2P structure in a 243 atoms cells, which we show in Fig. S4. We observe no difference in the predictions of the melting temperature, validating the 72-atoms structure as large enough.

Time convergence is also an important issue that must be addressed. In Fig. S5, we show the evolution of temperature as a function of time for different initial conditions close to the melting temperature, using the pyrite-type structure. The initial velocities are randomly assigned, so that they correspond to the a given temperature, leading to different values of instantaneous temperatures in the simulation. Homogeneous melting occures during the first 200 steps (40 fs) for the sample initiated at 26000 K , but the structure remains as a superheated solid when initiated at 23000 K (Fig. S5, left). The mean temperatures of the superheated solid and the final liquid are $T_{\text{sol}} = 9821$ K and $T_{\text{liq}} = 8815$ K, while the mean pressures are $P_{\text{sol}} = 375$ GPa and $P_{\text{liq}} = 391$ GPa. We observe that, whether we use 5000 steps (1 ps) or 30000 steps (6 ps), the mean temperature does not change, showing that 5000 steps of 0.2 fs is converged enough for our purposes.

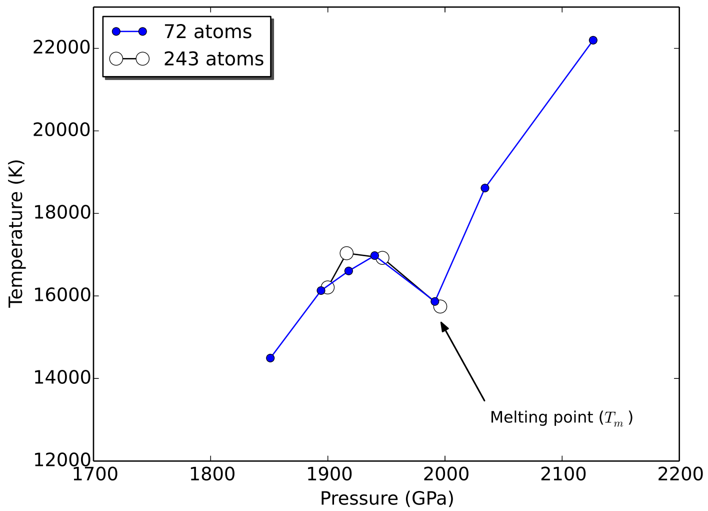

FIG. S4: Isochore for the Fe_2P structure using supercells of 72 and 243 atoms. No differences are appreciated in the prediction of the melting point.

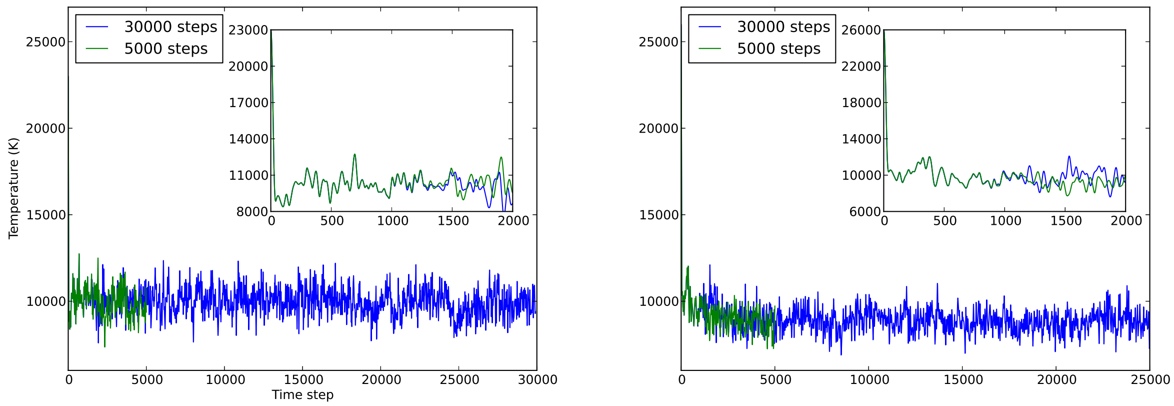

(a)$T_0 = 23000$ K. $\langle T \rangle = T_{\text{sol}} = 9821$ K (Solid branch)

(b)$T_0 = 26000$ K. $\langle T \rangle = T_{\text{liq}} = 8815$ K (Liquid branch)

FIG. S5: Four independent simulation runs for a volume $V = 5.06$ Å^3/atom ($\rho = 6.58$ g/cm^3) of the pyrite structure: two initiated at 23000 K (left) and two initiated at 26000 K (right).

S3. BAYESIAN ESTIMATION FOR THE Z-METHOD

We describe an isochoric curve (a Z-curve, in the following) by the four-tuple $(T_{LS}, T_m, C_v^S, C_v^L)$, where the last two parameters are the specific heat values for the solid and liquid branches, respectively. The energy of melting E_{LS} is not an independent parameter, as can be determined from

$$E_{LS} = \Phi_0 + C_v^S \cdot T_{LS}, \tag{S2}$$

where Φ_0 is the potential energy of the ideal crystalline structure.

In Bayesian estimation[31] we propose a probability distribution for the unknown parameters of a Z-curve given the actual (E_i, T_i) points obtained from n different microcanonical simulations. According to Bayes' theorem, this probability is given by

$$P(T_{LS}, T_m, C_v^S, C_v^L | \{E_i, T_i\}) = \frac{1}{\eta} P_0(T_{LS}, T_m, C_v^S, C_v^L) \prod_{j=1}^{n} P(E_j, T_j | T_{LS}, T_m, C_v^S, C_v^L). \tag{S3}$$

Here P_0 represents the *prior* probability for the Z-curve and η is just a normalization constant. This prior probability encodes what is known about the possible values of the parameters of the Z-curve before any actual data points are taken into account in the analysis. In our case, the prior is constructed simply by including consistency information that rejects impossible combinations of parameters which generate invalid Z-curves (for instance, we impose that $T_{LS} > T_m$).

The estimations of T_m with their associated error bars, were computed from this probability distribution in Eq. S3 using Monte Carlo Metropolis sampling[32].

The melting curve thus obtained, and fitted accordingly[33], corresponds to the blue line in Fig. 1 of the main manuscript.

---

* Electronic address: fgonzalez@lpmd.cl

1 Alfè, D., Cazorla, C. & Gillan, M. J. The kinetics of homogeneous melting beyond the limit of superheating. *Journal of Chemical Physics* **135**, 19 (2011).

2 Belonoshko, A., Skorodumova, N., Rosengren, A. & Johansson, B. Melting and critical superheating. *Physical Review B* **73**, 012201 (2006).

3 Belonoshko, A. *et al.* Properties of the fcc Lennard-Jones crystal model at the limit of superheating. *Physical Review B* **76**, 064121 (2007).

4 Belonoshko, A. *et al.* Molybdenum at High Pressure and Temperature: Melting from Another Solid Phase. *Physical Review Letters* **100**, 135701 (2008).

5 Belonoshko, A., Arapan, S., Martonak, R. & Rosengren, A. MgO phase diagram from first principles in a wide pressure-temperature range. *Physical Review B* **81**, 054110 (2010).

6 Belonoshko, A. & Rosengren, a. High-pressure melting curve of platinum from ab initio Z method. *Physical Review B* **85**, 174104 (2012).

7 Benazzouz, B. K., Zaoui, A. & Belonoshko, A. B. Determination of the melting temperature of kaolinite by means of the Z-method. *American Mineralogist* **98**, 1881–1885 (2013).

8 Bouchet, J., Bottin, F., Jomard, G. & Zérah, G. Melting curve of aluminum up to 300 GPa obtained through ab initio molecular dynamics simulations. *Physical Review B* **80**, 094102 (2009).

9 Burakovsky, L. *et al.* High-Pressure–High-Temperature Polymorphism in Ta: Resolving an Ongoing Experimental Controversy. *Physical Review Letters* **104**, 255702 (2010).

10 Burakovsky, L., Chen, S. P., Preston, D. L. & Sheppard, D. G. Z methodology for phase diagram studies: platinum and tantalum as examples. *Journal of Physics: Conference Series* **500**, 162001 (2014).

11 Davis, S., Belonoshko, A., Johansson, B., Skorodumova, N. V. & van Duin, A. C. T. High-pressure melting curve of hydrogen. *The Journal of chemical physics* **129**, 194508 (2008).

12 Davis, S., Belonoshko, A. B., Rosengren, A., van Duin, A. C. & Johansson, B. Molecular dynamics simulation of zirconia melting. *Central European Journal of Physics* **8**, 789–797 (2010).

13 Davis, S., Belonoshko, A., Johansson, B. & Rosengren, A. Model for diffusion at the microcanonical superheating limit from atomistic computer simulations. *Physical Review B* **84**, 064102 (2011).

14 Davis, S. & Gutiérrez, G. Bayesian inference as a tool for analysis of first-principles calculations of complex materials: an application to the melting point of Ti_2GaN. *Modelling and Simulation in Materials Science and Engineering* **21**, 075001 (2013).

15 Finney, A. R. & Rodger, P. M. Applying the z method to estimate temperatures of melting in structure ii clathrate hydrates. *Phys. Chem. Chem. Phys.* **13**, 19979–19987" (2011).

16 Li, D., Zhang, P., Yan, J. & Liu, H. Y. Melting curve of lithium from quantum molecular-dynamics simulations. *EPL (Europhysics Letters)* **95**, 56004 (2011).

17 Li, D., Zhang, P. & Yan, J. Ab initio molecular dynamics study of high-pressure melting of beryllium oxide. *Scientific reports* **4**, 4707 (2014).

18 Moriarty, J. A., Hood, R. Q. & Yang, L. H. Quantum-Mechanical Interatomic Potentials with Electron Temperature for Strong-Coupling Transition Metals. *Physical Review Letters* **108**, 036401 (2012).

19 Sun, J., Martinez-Canales, M., Klug, D. D., Pickard, C. J. & Needs, R. J. Stable All-Nitrogen Metallic Salt at Terapascal Pressures. *Physical Review Letters* **111**, 175502 (2013).

20 Tsuchiya, T. & Tsuchiya, J. Prediction of a hexagonal SiO_2 phase affecting stabilities of $MgSiO_3$ and $CaSiO_3$ at multimegabar pressures. *Proceedings of the National Academy of Sciences of the United States of America* **108**, 1252–5 (2011).

21 Wu, S. *et al.* Identification of post-pyrite phase transitions in SiO_2 by a genetic algorithm. *Physical Review B* **83**, 184102 (2011).

[22] Umemoto, K., Wentzcovitch, R. M. & Allen, P. B. Dissociation of $MgSiO_3$ in the cores of gas giants and terrestrial exoplanets. *Science (New York, N.Y.)* **311**, 983–6 (2006).
[23] González-Cataldo, F., Wilson, H. F. & Militzer, B. Ab Initio Free Energy Calculations of the Solubility of Silica in Metallic Hydrogen and Application To Giant Planet Cores. *The Astrophysical Journal* **787**, 79 (2014).
[24] Wahl, S. M., Wilson, H. F. & Militzer, B. Solubility of Iron in Metallic Hydrogen and Stability of Dense Cores in Giant Planets. *The Astrophysical Journal* **773**, 95 (2013).
[25] Wilson, H. & Militzer, B. Solubility of Water Ice in Metallic Hydrogen: Consequences for Core Erosion in Gas Giant Planets. *The Astrophysical Journal* **745**, 54 (2012).
[26] Militzer, B. & Hubbard, W. B. Ab Initio Equation of State for Hydrogen-Helium Mixtures With Recalibration of the Giant-Planet Mass-Radius Relation. *The Astrophysical Journal* **774**, 148 (2013).
[27] Correa, A., Benedict, L., Young, D., Schwegler, E. & Bonev, S. A. First-principles multiphase equation of state of carbon under extreme conditions. *Physical Review B* **78**, 024101 (2008).
[28] Kresse, G. & Furthmüller, J. Efficient iterative schemes for ab initio total-energy calculations using a plane-wave basis set. *Phys. Rev. B* **54**, 11169 (1996).
[29] Blochl, P. E. Projector augmented-wave method. *Phys. Rev. B* **50**, 17953 (1994).
[30] Perdew, J. P., Burke, K. & Ernzerhof, M. Generalized gradient approximation made simple. *Phys. Rev. Lett.* **77**, 3865 (1996).
[31] Sivia, D. S. & Skilling, J. *Data Analysis: A Bayesian Tutorial* (Oxford: Oxford University Press, 2006).
[32] Gamerman, D. & Lopes, H. F. *Markov Chain Monte Carlo: Stochastic Simulation for Bayesian Inference* (Taylor and Francis, 2006).
[33] Kechin, V. Melting curve equations at high pressure. *Physical Review B* **65**, 052102 (2001).
